# Association Between Cardiovascular Autonomic Function and Temporomandibular Disorders

**DOI:** 10.1111/joor.14051

**Published:** 2025-06-09

**Authors:** Niklas Kakko, Auli Suominen, Atte Somero, Mikko Tulppo, Satu Lahti, Vesa Pohjola, Mika Ogawa, Kirsi Sipilä

**Affiliations:** ^1^ Research Unit of Population Health, Faculty of Medicine University of Oulu Oulu Finland; ^2^ Department of Community Dentistry University of Turku Turku Finland; ^3^ Research Unit of Biomedicine and Internal Medicine University of Oulu Oulu Finland; ^4^ Medical Research Center Oulu Oulu University Hospital and University of Oulu Oulu Finland; ^5^ Systemic Approaches to Improve Cardiometabolic and Brain Health During Lifespan (SYS‐LIFE) Co‐Funded by University of Turku and the European Union Turku Finland

**Keywords:** autonomic nervous system activity, cardiovascular autonomic function, population‐based, temporomandibular disorders

## Abstract

**Background:**

Studies have shown that elevated stress levels associate with TMD‐related pain, which suggests that alterations in autonomic tone may contribute to this pain condition.

**Objective:**

The aim of the study was to evaluate the sex‐specific associations between autonomic nervous system (ANS) activity and TMD pain‐related diagnoses in a population‐based study.

**Methods:**

The study was part of the Northern Finland Birth Cohort 1966. Of the cohort members, 1964 (62.3% of those invited to oral health examination) were clinically examined as part of the 46‐year follow‐up. ANS activity was assessed by means of heart rate variability (HRV) and baroreflex sensitivity (BRS). A total of 5 TMD diagnoses were based on the modified protocol of DC/TMD (Diagnostic Criteria for TMD). Of those, pain‐related diagnoses, i.e., myalgia and arthralgia, were used. In logistic regression analyses stratified by sex assigned at birth, potential confounders, i.e., education, body mass index, and number of body pain sites, were considered.

**Results:**

Those with TMD myalgia (*n* = 97) or arthralgia diagnoses (*n* = 102) had lower values of BRS while standing when adjusted for covariates among females (for myalgia OR 0.847, 95% Cl 0.744–0.964, *p* = 0.012) and for arthralgia (OR 0.871, 95% Cl 0.775–0.970, *p* = 0.021).

**Conclusion:**

The results suggest that lowered baroreflex sensitivity, indicating increased sympathetic tone, associates with TMD pain, at least to some extent, in females. These findings refer to the association of stress response with TMD.

## Graphical abstract

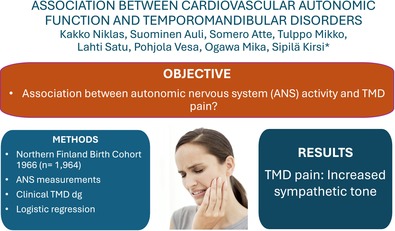

## 1 Introduction

Temporomandibular disorders (TMD) is a collective term for pain and dysfunction in masticatory muscles, temporomandibular joints (TMJs), and associated structures [1]. Pain in the facial and jaw area is a common symptom of TMD [1]. The clinical diagnostics of TMD are based on symptom reporting and clinical signs detected in the examination. Of the clinical TMD sub‐diagnoses, myalgia refers to masticatory muscle pain and arthralgia to TMJ pain [1]. Approximately 34%–38% of the population have clinical TMD signs [2, 3, 4], whereas in a large, unselected population of the Northern Finland Birth Cohort 1966 (NFBC1966) the prevalence of diagnosed TMD was 4%–8%, the prevalence of myalgia was 5.0%, and that of arthralgia was 5.3% [5]. The prevalence levels of TMD are 1.5–2.0 times higher among women than among men [5, 6].

The aetiology of TMD is multifactorial; psychosocial factors, such as stress, depression, somatisation, and anxiety, play an important role in the background of TMD [7, 8]. A strong association between TMD‐related pain and higher stress levels has been reported, and this may be explained by changes in autonomic nervous system (ANS) activity, which may contribute to pain [9].

ANS regulates cardiovascular function by afferent and efferent autonomic neural pathways [10]. ANS activity can be estimated by heart rate variability (HRV) and baroreflex sensitivity (BRS) [11, 12, 13]. HRV is the variation in contiguous heartbeat‐to‐beat times and is an accurate method of measuring parasympathetic and sympathetic nervous system activity [11]. HRV can be separated into its component rhythms that operate in different frequency ranges by Fast Fourier Transformation. LF (low frequency) band and HF (high frequency) band are mostly used to reflect the ANS activity. Of these, LF power indicates both parasympathetic and sympathetic nervous system activity, whereas HF band reflects parasympathetic activity [11, 14]. BRS, as an indicator for autonomic input to the sinus node, chiefly measures how vagal activity is responding to stressors. It is measured by changes in R–R intervals, which are the result of induced changes in blood pressure. BRS is generally measured by defining the magnitude of induced bradycardia in response to a pressor. It decreases with age from young adulthood and is reduced in patients with heart failure or hypertension [12, 13].

Although ANS activity most importantly regulates physiological stability, it also regulates and modifies the perception of pain [15, 16]. Many studies have recognised a connection between pain and ANS activity [17, 18]. Associations of the dysregulation of ANS activity have also been found with chronic widespread pain (CWP) [19] and musculoskeletal pain [20]. As TMD pain is included in musculoskeletal pains, ANS activity may have a role in TMD‐related pain.

There are not many studies about the connection between ANS activity and TMD. Based on the OPPERA (Orofacial Pain: Prospective Evaluation and Risk Assessment) study, TMD cases showed elevated HR, reduced HRV [21], as well as reduced BRS compared to non‐TMD controls [22]. These results are also in line with a study on TMD patients [23]. These findings suggest that TMD patients may have a tendency towards higher sympathetic tone versus parasympathetic tone. However, further studies, especially population‐based studies, are needed to investigate the association between ANS activity and TMD pain. The aim of the present study was to evaluate the association between the cardiovascular autonomic function and TMD pain‐related diagnoses in the NFBC1966 study and to investigate differences in these associations according to sex assigned at birth.

## 2 Materials and Methods

The study population is part of NFBC1966, including subjects born in 1966 in the two Northern provinces of Oulu and Lapland (*n* = 12 231) [24, 25]. The cohort has been followed from prenatal age with clinical data collections and regular questionnaires. In the latest follow‐up in 2012–2014, subjects with a known address (*n* = 10 321) received postal questionnaires to be filled in at home, including questions about dental health, health‐related behaviour, and general well‐being. Of the participants, 6868 responded to the postal questionnaire.

The basic clinical health examinations, including cardiovascular autonomic activity, were performed by three research nurse teams in 36 towns all over Finland. Cardiovascular autonomic activity was evaluated by means of BRS and HRV parameters at the 46‐year clinical examinations (*n* = 5861) as reported by Oura et al. [20]. Six‐minute data was recorded for HRV and BRS analyses using a HR monitor (RS800CX, Polar Electro Oy, Kempele, Finland) and a standard lead‐II ECG (Cardiolife Nihon Kohden, Tokyo, Japan). The first 3 min were recorded in a sitting position and the 3 min immediately after that in a standing position. The HRV was recorded during the first 150 s in a sitting position and during the last 150 s in a standing position. Examinations were preceded by at least a 1‐min stabilisation period. BRS was analysed by processing the blood pressure, ECG, and ventilation rate data with the University of Oulu Biosignal Processing team's customised Matlab software.

Additionally, a subpopulation (*n* = 3150) living within 100 km of the city of Oulu was invited to a field study, and a total of 1964 individuals participated in the clinical examination. The final number of subjects was 1962 because two participants declined the use of their data. The examination of oral health was accomplished at the beginning of the clinical health examination. The study followed the principles of the Declaration of Helsinki. Participants' rights have been protected by an appropriate Institutional Review Board. Written informed consent was given by all participants. The Ethics Committee of the Northern Ostrobothnia Hospital District approved the research (74/2011) [24, 25].

The TMD symptom questionnaire and clinical TMD examination were performed by the modified protocol of DC/TMD (Diagnostic Criteria for Temporomandibular Disorders) [26, 27]. In connection with the clinical examination, the following questions on TMD pain symptoms were asked:

1. Have you had pain in areas of your face, jaws, temples, ears, or behind your ears during the prior 30 days? (with options yes/no)
2. During the prior 30 days, have you felt pain that was modified by jaw movement, function, parafunction, or being at rest? (with options yes/no)

The examination of TMD was accomplished by five calibrated dentists. Before the study, the investigators were trained by experienced specialised dentists to ensure the repeatability of the clinical examination. Inter‐examiner agreement was also determined regularly during the study by a senior dentist serving as a gold standard.

The clinical TMD examination included measurement of maximum mouth opening without any assistance by the examiner and lateral and protrusive movements. In maximum assisted opening, the jaw was actively pushed on by the examiner. The patients were asked if they felt “familiar” pain in any movements, defined as pain that was “similar” to or “like” the pain they had previously had in the same location during the preceding 30 days. Familiar pain in the area of the TMJ and masticatory muscles was registered.

The temporal muscles were palpated bilaterally at the anterior, middle, and posterior regions and the masseter muscles were palpated bilaterally at the origo, profunda, and insertion regions. Palpation of the temporal and masseter muscles was done by applying a pressure of 1.0 kg (2–3 pounds). Palpation of the TMJ was done by applying a pressure of 1.0 kg (2–3 pounds) around the TMJ pole, and 0.5 kg (1 pound) for the lateral pole of the TMJ. Palpation forces were calibrated with a digital postage scale. A total of 1895 subjects (95.5%) both filled in the symptom questionnaire and attended the clinical examination.

The sub‐diagnoses of TMD used in the present study were myalgia and arthralgia, based on the following criteria:

– Myalgia: Pain during the previous 30 days in areas of the jaws, face, ears, behind the ears, or temples, pain modified by movement, and familiar pain on palpation of the masticatory muscles and/or during jaw movements [2].
– Arthralgia: Pain during the previous 30 days in areas of the jaws, face, ears, behind the ears or temples, pain modified by movement, and familiar pain on palpation of the TMJ and/or jaw movements [2].

Information of potential confounders that have been shown to associate with TMD [5] or ANS activity [28, 29, 30] was collected from the postal questionnaire. These were smoking, education, body mass index (BMI), diabetes mellitus type I, diabetes mellitus type II and use of beta‐blockers. Smoking was categorised as follows: (1) smoker (daily smoker and occasional smoker), (2) former smoker (currently non‐smoker, previously smoked daily for at least 1 year), and (3) never smoker (never smoked or currently non‐smoker but had previously smoked daily for less than a year or had left the item unfilled). The level of education was classified as ‘basic education’ (no high school graduation/academic vocational qualification), ‘secondary education’ (vocational school or high school graduation) and ‘higher education’ (university degree/polytechnic or comparable school graduation). BMI was calculated by using clinically measured height and weight. BMI was classified as three categories based on classifications set by the World Health Organisation (WHO): (1) underweight (under 18.5 kg/m^2^) and normal weight (18.5–24.9 kg/m^2^), (2) overweight (25–29.9 kg/m^2^) and (3) obesity (30 kg/m^2^ and over). Due to the low number of underweight participants with TMD diagnoses, they were included in category 1.

Other body pains were inquired by a question: ‘Have you had any aches or pains in the following areas of your body within the last 12 months (yes/no)? (1) neck, (2) shoulders, (3) arms/elbows, (4) wrists/hands, (5) low back, (6) hips, (7) knees, (8) ankles/feet’. The question was accompanied with a body pain drawing for identifying these anatomical areas. Each positive response was followed by a subsequent question: ‘How often have you had aches or pains in this area during the last 12 months? (1) On 1–7 days, (2) On 8–30 days, (3) On more than 30 days but not daily, (4) Daily’. A sum score of body pain (range 0–32) was calculated, indicating the number of body pain sites and the frequency of pains.

### 2.1 Statistical Analyses

Characteristics of the study sample were described by calculating means and standard deviations of continuous variables and the frequencies and percentages of the categorical variables. Associations of sex with TMD diagnoses, ANS variables, and covariates were calculated by *t*‐tests (variables with normal distribution) and Mann–Whitney tests (variables with skewed distribution). The distribution of variables was analysed by skewness and kurtosis statistic values (normal distribution −1 < *n* < 1). Associations of ANS variables with TMD diagnoses were evaluated using parametric and non‐parametric methods, stratified by sex assigned at birth. Associations of TMD diagnoses with possible confounders were evaluated using chi‐square test and Mann–Whitney test. In the logistic regression analyses, the dependent variables were TMD myalgia and arthralgia (categorised as diagnosis vs. no diagnosis), and ANS activity parameters (as continuous variable) were included as independent variables. Those covariates (education, BMI and sum score of body pain) that were significantly associated with TMD diagnoses (myalgia and arthralgia) were included. Secondary education and normal weight were used as reference in covariates in the logistic regression analyses. IBM SPSS Statistics (version 29.0.0.0) was used for analyses. The *p*‐values of < 0.05 were considered indicative of statistical differences.

## 3 Results

The basic characteristics of the study sample by sex are shown in Table 1. Females had 3.4 times more often diagnoses of myalgia and 4.3 times more often of arthralgia than males. There were statistically significant sex differences in almost all mean HRV and BRS variables and in covariates (Table 1). Females had higher seated lnrMSSD, seated lnHFfft, standing HR, standing SDNN, and standing lnHFfft than males. In contrast, males had higher seated lnLFfft, seated LF/HFfft, seated SBP, seated DBP, seated SPBV, seated BRS, standing lnLFfft, standing LF/HFfft, standing SBP, standing DBP, standing SBPV, and standing BRS (Table 1). Males were statistically significantly more often overweight, had diabetes, were current smokers, and were single/lived alone (Table 1). However, statistically significantly more females were using beta‐blockers, had higher education, and were married/cohabiting (Table 1).

**TABLE 1:** Basic characteristics of the study sample including in the Northern Finland 1966 Birth Cohort. The combined arthralgia/myalgia group is a subset of the total diagnosed with a TMD. Total numbers variate due to missing data.

Characteristic	Total *n*	Females	Males	*p*
TMD		*n* (%)	*n* (%)	
Myalgia diagnosis	1948	77 (7.4)	20 (2.2)	< 0.001^a^
Arthralgia diagnosis	1932	85 (8.2)	17 (1.9)	< 0.001^a^
Myalgia and arthralgia dg	1932	61 (5.9)	13 (1.5)	< 0.001^a^
ANS seated		Mean (SD)	Mean (SD)	
HR, seated	5715	72.19 (10.68)	71.61 (12.08)	0.058^b^
SDNN, seated	5715	38.54 (17.23)	38.43 (18.34)	0.820^b^
lnrMSSD, seated	5715	3.14 (0.61)	2.99 (0.64)	< 0.001^b^
lnLFfft, seated	5715	5.68 (1.01)	5.85 (1.04)	< 0.001^b^
lnHFfft, seated	5715	5.44 (1.25)	5.04 (1.29)	< 0.001^b^
LF/HFfft, seated	5713	1.90 (2.10)	3.23 (3.36)	< 0.001^c^
SBP (mmHg), seated	2651	116.21 (16.63)	122.57 (14.11)	< 0.001^b^
DBP (mmHg), seated	2651	69.48 (9.39)	73.21 (8.23)	< 0.001^b^
SBPV (mmHg^2^), seated	2651	7.50 (5.39)	7.94 (5.70)	0.026^c^
BRS (ms/mmHg), seated	2650	6.86 (6.18)	7.58 (6.58)	< 0.001^c^
ANS standing				
HR, standing	5692	83.02 (12.41)	81.82 (13.36)	0.001^b^
SDNN, standing	5692	31.52 (13.45)	31.00 (15.99)	< 0.001^b^
lnrMSSD, standing	5692	2.54 (0.62)	2.52 (0.64)	0.219^b^
lnLFfft, standing	5692	5.28 (0.99)	5.57 (1.10)	< 0.001^b^
lnHFfft, standing	5692	4.30 (1.28)	4.15 (1.30)	< 0.001^b^
LF/HFfft, standing	5685	3.75 (3.68)	5.78 (5.19)	< 0.001^c^
SBP (mmHg), standing	2632	114.36 (16.66)	121.15 (14.32)	< 0.001^b^
DBP (mmHg), standing	2632	72.04 (9.42)	74.94 (8.48)	< 0.001^b^
SBPV (mmHg^2^), standing	2632	10.32 (7.41)	12.61 (9.09)	< 0.001^c^
BRS (ms/mmHg), standing	2626	4.78 (4.18)	5.29 (4.71)	< 0.001^c^
Covariates		*n* (%)	*n* (%)	
Body mass index	5765			
Underweight		23 (0.7)	6 (0.2)	< 0.001^a^
Normal weight		1458 (45.3)	760 (30.0)	
Over weight		1053 (32.7)	1214 (47.9)	
Obesity		683 (21.2)	553 (21.8)	
Diabetes mellitus				
type I	6688	15 (0.4)	30 (1.0)	0.006^a^
type II	6671	101 (2.8)	89 (2.9)	0.006^c^
Beta‐blocker usage	6760	256 (7.0)	161 (5.2)	0.003^a^
Smoking	7146			
Smoking (current)		810 (21.1)	894 (27.1)	< 0.001^a^
Smoking (former)		748 (19.4)	793 (24.0)	
Smoking (non‐smoker)		2289 (59.5)	1612 (48.9)	
Education	7146			
Basic		274 (7.1)	354 (10.7)	< 0.001^a^
Secondary		1104 (28.7)	1508 (45.7)	
Higher		2469 (54.7)	1437 (43.6)	
Marital status	6805			
Married/cohabiting		3318 (89.6)	2613 (84.2)	< 0.001^a^
Single/living alone		385 (10.4)	489 (15.8)	

Abbreviations: BMI, body mass index; BRS, baroreflex sensitivity; DBP, diastolic blood pressure; DM, diabetes mellitus; HFfft, high frequency power of R–R intervals oscillations; HR, heart rate; LFfft, low frequency power of R–R intervals oscillations; rMSSD, root mean square of successive differences in R–R intervals; SBP, systolic blood pressure; SBPV, systolic blood pressure variation; SD, standard deviation; SDNN, standard deviation of normal‐to‐normal R–R interval.

^a^ *T*Chi‐Square test.

^b^ T‐ *T*est.

^c^ Mann‐Whitney Test.

The associations of TMD myalgia and arthralgia with the potential confounders are presented in Table 2. The presence of myalgia was associated significantly with BMI, education, and the sum score of body pain. The associations between ANS variables and TMD myalgia in the total sample are presented in Table 3. Those with myalgia had significantly higher values of HR seated, HR standing, and lower values of SDNN standing, lnLFfft seated, lnLFfft standing, LF/HFfft standing, BRS seated, and BRS standing. When stratified by sex, many statistically significant differences disappeared. Among females, those with a myalgia diagnosis had higher HR seated and lower lnLFfft standing and BRS seated. Among males, there were no statistically significant differences in ANS variables between those with vs. without TMD myalgia. Table 4 shows associations between ANS variables and TMD arthralgia in the total sample. Those with an arthralgia diagnosis had significantly lower SDNN standing, lnLFfft standing, LF/HFfft seated, LF/HFfft standing, and BRS standing compared to those without arthralgia. When stratified by sex, females with arthralgia had significantly lower BRS standing than females without this diagnosis. Among males, those with arthralgia had higher SBP seated, DBP seated, SBP standing, and SBPV seated compared to those without arthralgia.

**TABLE 2:** Differences in covariates between those with TMD myalgia/arthralgia diagnosis vs. without diagnosis among the total sample of subjects included in the Northern Finland Birth 1966 cohort. The number of diagnoses varies due to missing data.

	Myalgia dg *n* (%)	No myalgia dg *n* (%)	*p*
Body mass index			
Underweight	1 (1.1)	5 (0.3)	
Normal weight	34 (36.6)	710 (39.0)	**0.049** ^**a**^
Over weight	29 (31.2)	722 (39.7)	
Obesity	29 (31.2)	382 (21.0)	
Diabetes mellitus			
Type I	0 (0)	18 (1.0)	0.355^a^
Type II	3 (3.5)	47 (2.7)	0.636^a^
Beta‐blocker usage	4 (4.7)	96 (5.4)	0.781^a^
Smoking			
Smoking (current)	23 (24.5)	382 (20.8)	
Smoking (former)	28 (29.8)	428 (23.3)	0.200^a^
Smoking (non‐smoker)	43 (45.7)	1025 (55.8)	
Education			
Basic	12 (12.8)	109 (5.9)	**0.021** ^**a**^
Secondary	29 (30.9)	687 (37.4)	
Higher	53 (56.4)	1039 (56.6)	
Marital status			
Married/cohabiting	79 (91.9)	1557 (87.3)	0.209^a^
Single/living alone	7 (8.1)	227 (12.7)	
Mean of sum score of body pain (SD)	12.2 (6.9)	7.5 (5.5)	**< 0.001** ^**b**^

	Arthralgia dg *n* (%)	No arthralgia dg *n* (%)	
Body mass index			
Underweight	1 (1.0)	5 (0.3)	
Normal weight	44 (44.0)	702 (38.7)	
Over weight	29 (29.0)	722 (39.8)	0.108^a^
Obesity	26 (26.0)	386 (21.3)	
Diabetes mellitus			
Type I	0 (0.0)	18 (1.0)	0.329^a^
Type II	1 (1.1)	49 (2.8)	0.319^a^
Beta‐blocker usage	5 (5.4)	96 (5.4)	0.986^a^
Smoking			
Smoking (current)	22 (21.6)	385 (21.0)	0.315^a^
Smoking (former)	29 (28.4)	427 (23.3)	
Smoking (non‐smoker)	51 (50.0)	1018 (55.6)	
Education			
Basic	13 (12.7)	108 (5.9)	**0.003** ^**a**^
Secondary	26 (25.5)	690 (37.7)	
Higher	63 (61.8)	1032 (56.4)	
Marital status			
Married/cohabiting	85 (89.5)	1554 (87.4)	0.552^a^
Single/living alone	10 (10.5)	224 (12.6)	
Mean of sum score of body pain (SD)	11.8 (6.8)	7.5 (5.6)	**< 0.001** ^**b**^

^a^ Chi Square test.

^b^ Mann‐Whitney test.

**TABLE 3:** Differences in variables of autonomic nervous system activity between those with TMD myalgia diagnosis vs. without diagnosis among the total sample of subjects included in the Northern Finland Birth 1966 cohort (*n* = 1948).

Total sample	Myalgia dg (*n* = 97), Mean (SD)	No myalgia dg (*n* = 1851), Mean (SD)	*p*
HR, seated	77.80 (9.90)	75.38 (11.71)	**0.047** ^**a**^
SDNN, seated	29.44 (13.91)	32.37 (14.80)	0.058^a^
lnrMSSD, seated	2.79 (0.49)	2.86 (0.63)	0.182^a^
lnLFfft, seated	5.26 (1.01)	5.48 (1.09)	**0.049** ^**a**^
lnHFfft, seated	4.82 (1.02)	4.87 (1.33)	0.677^a^
LF/HFfft, seated	2.45 (2.72)	2.86 (3.27)	0.061^b^
SBP (mmHg), seated	115.46 (14.85)	116.84 (15.41)	0.394^a^
DBP (mmHG), seated	68.67 (7.93)	69.66 (8.85)	0.288^a^
SBPV, seated	7.08 (6.13)	7.63 (7.86)	0.828^b^
BRS, seated	6.27 (2.69)	7.34 (4.07)	**0.043** ^**b**^
HR, standing	89.16 (12.30)	86.07 (13.26)	**0.026** ^**a**^
SDNN, standing	24.92 (9.74)	28.29 (13.15)	**0.013** ^**a**^
lnrMSSD, standing	2.27 (0.57)	2.37 (0.63)	0.153^a^
lnLFfft, standing	4.85 (1.09)	5.18 (1.10)	**0.004** ^**a**^
lnHFfft, standing	3.80 (1.18)	3.92 (1.37)	0.427^a^
LF/HFfft, standing	4.12 (3.75)	5.01 (4.82)	**0.033** ^**b**^
SBP (mmHg), standing	113.66 (14.18)	115.41 (15.65)	0.290^a^
DBP (mmHg), standing	70.66 (7.68)	71.84 (8.93)	0.208^a^
SBPV, standing	11.45 (12.50)	11.40 (11.50)	0.477^b^
BRS, standing	4.22 (2.18)	5.09 (3.03)	**0.008** ^**b**^
Females			
HR, seated	78.46 (10.15)	75.87 (11.05)	**0.048** ^**a**^
SDNN, seated	29.64 (14.74)	31.90 (13.73)	0.167^a^
lnrMSSD, seated	2.82 (0.49)	2.91 (0.61)	0.166^a^
lnLFfft, seated	5.20 (1.02)	5.37 (1.05)	0.155^a^
lnHFfft, seated	4.93 (1.02)	5.00 (1.32)	0.534^a^
LF/HFfft, seated	2.04 (2.47)	2.23 (2.38)	0.273^b^
SBP (mmHg), seated	113.03 (13.41)	113.29 (16.12)	0.890^a^
DBP (mmHg), seated	67.20 (7.15)	67.80 (9.23)	0.490^a^
SBPV, seated	6.37 (4.88)	7.54 (7.97)	0.606^b^
BRS, seated	6.39 (2.87)	7.01 (3.68)	0.356^b^
HR, standing	89.63 (12.37)	86.70 (12.89)	0.054^a^
SDNN, standing	24.68 (9.65)	26.92 (11.46)	0.095^a^
lnrMSSD, standing	2.24 (0.57)	2.36 (0.61)	0.107^a^
lnLFfft, standing	4.73 (1.06)	5.01 (1.00)	**0.020** ^**a**^
lnHFfft, standing	3.79 (1.22)	3.94 (1.37)	0.335^a^
LF/HFfft, standing	3.55 (3.04)	4.12 (4.22)	0.295^b^
SBP (mmHg), standing	112.16 (14.10)	111.69 (16.27)	0.809^a^
DBP (mmHg), standing	69.78 (7.50)	70.46 (9.33)	0.461^a^
SBPV, standing	10.16 (9.85)	10.10 (10.92)	0.921^b^
BRS, standing	4.04 (1.85)	4.91 (2.89)	**0.024** ^**b**^
Males			
HR, seated	75.13 (8.57)	74.84 (12.37)	0.918^a^
SDNN, seated	28.63 (10.15)	32.89 (15.88)	0.245^a^
lnrMSSD, seated	2.65 (0.44)	2.80 (0.64)	0.284^a^
lnLFfft, seated	5.50 (0.96)	5.60 (1.12)	0.702^a^
lnHFfft, seated	4.40 (0.95)	4.72 (1.34)	0.300^a^
LF/HFfft, seated	4.12 (3.09)	3.54 (3.90)	0.184^b^
SBP (mmHg), seated	125.19 (16.66)	120.71 (13.58)	0.158^a^
DBP (mmHg), seated	74.57 (8.36)	71.68 (7.93)	0.116^a^
SBPV, seated	9.92 (9.30)	7.72 (7.75)	0.214^b^
BRS, seated	5.76 (4.43)	5.76 (1.77)	0.065^b^
HR, standing	87.25 (12.19)	85.39 (13.63)	0.555^a^
SDNN, standing	25.89 (10.31)	29.78 (14.64)	0.250^a^
lnrMSSD, standing	2.42 (0.56)	2.38 (0.64)	0.810^a^
lnLFfft, standing	5.35 (1.07)	5.37 (1.17)	0.950^a^
lnHFfft, standing	3.88 (1.01)	3.89 (1.38)	0.964^a^
LF/HFfft, standing	6.46 (5.30)	5.97 (5.23)	0.729^b^
SBP (mmHg), standing	120.37 (12.86)	119.45 (13.88)	0.786^a^
DBP (mmHg), standing	74.56 (7.46)	73.34 (8.23)	0.546^a^
SBPV, standing	17.24 (20.00)	12.80 (11.94)	0.725^b^
BRS, standing	5.02 (3.22)	5.29 (3.17)	0.619^b^

Abbreviations: BRS, baroreflex sensitivity; DBP, diastolic blood pressure; HFfft, high frequency power of R–R interval oscillations; HR, heart rate; LFfft, low frequency power of R–R interval oscillations; rMSSD, root mean square of successive differences in R–R intervals; SBP, systolic blood pressure; SBPV, systolic blood pressure variation; SDNN, standard deviation of normal‐to‐normal R–R interval.

^a^ *T*‐test.

^b^ Mann–Whitney Test.

**TABLE 4:** Differences in variables of autonomic nervous system activity (mean, standard deviation), between those with TMD arthralgia diagnosis vs. without diagnosis among the total sample of subjects included in the Northern Finland Birth 1966 cohort (*n* = 1932).

Total sample	Arthralgia dg (*n* = 102)	No arthralgia dg (*n* = 1830)	*p*
HR, seated	76.57 (10.03)	75.35 (11.64)	0.307^a^
SDNN, seated	31.95 (15.72)	32.33 (14.72)	0.802^a^
lnrMSSD, seated	2.88 (0.54)	2.86 (0.62)	0.745^a^
lnLFfft, seated	5.36 (1.04)	5.48 (1.09)	0.273^a^
lnHFfft, seated	4.98 (1.12)	4.87 (1.33)	0.316^a^
LF/HFfft, seated	2.36 (2.76)	2.86 (3.27)	**0.011** ^**b**^
SBP (mmHg), seated	115.43 (15.61)	116.85 (15.34)	0.370^a^
DBP (mmHg), seated	68.62 (8.75)	69.63 (8.80)	0.267^a^
SBPV, seated	7.39 (6.67)	7.63 (7.87)	0.899^b^
BRS, seated	6.82 (3.38)	7.32 (4.05)	0.449^b^
HR, standing	87.33 (12.83)	86.05 (13.16)	0.342^a^
SDNN, standing	25.33 (10.38)	28.36 (13.15)	**0.024** ^**a**^
lnrMSSD, standing	2.32 (0.60)	2.37 (0.62)	0.459^a^
lnLFfft, standing	4.90 (1.10)	5.19 (1.10)	**0.012** ^**a**^
lnHFfft, standing	3.89 (1.25)	3.92 (1.36)	0.828^a^
LF/HFfft, standing	3.99 (3.54)	5.02 (4.83)	**0.010** ^**b**^
SBP (mmHg), standing	113.55 (14.37)	115.43 (15.63)	0.246^a^
DBP (mmHg), standing	70.65 (8.25)	71.82 (8.88)	0.205^a^
SBPV, standing	11.71 (12.14)	11.41 (11.54)	0.970^b^
BRS, standing	4.28 (2.20)	5.10 (3.03)	**0.010** ^**b**^
Females			
HR, seated	76.70 (10.08)	75.94 (11.02)	0.544^a^
SDNN, seated	31.52 (15.74)	31.82 (13.65)	0.850^a^
lnrMSSD, seated	2.89 (0.56)	2.90 (0.60)	0.860^a^
lnLFfft, seated	5.27 (1.00)	5.37 (1.06)	0.412^a^
lnHFfft, seated	5.04 (1.15)	5.00 (1.31)	0.768^a^
LF/HFfft, seated	1.98 (2.49)	2.23 (2.38)	0.171^b^
SBP (mmHg), seated	112.34 (13.29)	113.42 (16.14)	0.554^a^
DBP (mmHg), seated	67.12 (7.64)	67.82 (9.21)	0.499^a^
SBPV, seated	6.33 (4.98)	7.56 (7.99)	0.395^b^
BRS, seated	6.93 (3.45)	6.97 (3.65)	0.756^b^
HR, standing	87.60 (13.02)	86.76 (12.79)	0.565^a^
SDNN, standing	24.88 (10.28)	26.98 (11.42)	0.104^a^
lnrMSSD, standing	2.29 (0.61)	2.36 (0.61)	0.324^a^
lnLFfft, standing	4.79 (1.10)	5.01 (1.00)	0.064^a^
lnHFfft, standing	3.86 (1.31)	3.94 (1.36)	0.578^a^
LF/HFfft, standing	3.70 (3.46)	4.10 (4.20)	0.233^b^
SBP (mmHg), standing	111.12 (12.86)	111.83 (16.39)	0.703^a^
DBP (mmHg), standing	69.52 (7.44)	70.49 (9.34)	0.270^a^
SBPV, standing	10.40 (9.66)	10.04 (10.94)	0.519^b^
BRS, standing	4.26 (2.30)	4.91 (2.87)	**0.048** ^**b**^
Males			
HR, seated	75.88 (10.09)	74.71 (12.25)	0.705^a^
SDNN, seated	34.19 (15.89)	32.88 (15.83)	0.744^a^
lnrMSSD, seated	2.80 (0.45)	2.80 (0.64)	0.972^a^
lnLFfft, seated	5.81 (1.12)	5.60 (1.11)	0.463^a^
lnHFfft, seated	4.68 (0.96)	4.72 (1.33)	0.914^a^
LF/HFfft, seated	4.38 (3.27)	3.55 (3.91)	0.195^b^
SBP (mmHg), seated	131.44 (17.29)	120.57 (13.47)	**0.002** ^**a**^
DBP (mmHg), seated	76.45 (10.13)	71.60 (7.86)	**0.015** ^**a**^
SBPV, seated	12.92 (10.78)	7.71 (7.73)	**0.011** ^**b**^
BRS, seated	6.28 (3.01)	7.71 (4.43)	0.172^a^
HR, standing	85.95 (12.07)	85.28 (13.52)	0.845^a^
SDNN, standing	27.69 (10.87)	29.85 (14.65)	0.557^a^
lnrMSSD, standing	2.51 (0.52)	2.38 (0.64)	0.445^a^
lnLFfft, standing	5.46 (0.98)	5.38 (1.17)	0.777^a^
lnHFfft‐standing	4.08 (0.88)	3.90 (1.35)	0.597^a^
LF/HFfft, standing	5.46 (3.73)	6.02 (5.26)	0.952^b^
SBP (mmHg), standing	126.80 (15.39)	119.33 (13.74)	**0.038** ^**a**^
DBP (mmHg), standing	76.82 (9.91)	73.26 (8.13)	0.094^a^
SBPV, standing	18.89 (20.12)	12.88 (12.00)	0.250^b^
BRS, standing	4.37 (1.55)	5.31 (3.18)	0.371^b^

Abbreviations: BRS, baroreflex sensitivity; DBP, diastolic blood pressure; HFfft, high frequency power of R–R intervals oscillations; HR, heart rate; LFfft, low frequency power of R–R intervals oscillations; rMSSD, root mean square of successive differences in R–R intervals; SBP, systolic blood pressure; SBPV, systolic blood pressure variation; SDNN, standard deviation of normal‐to‐normal R–R‐interval.

^a^ *T*‐test.

^b^ Mann–Whitney Test.

The logistic regression analyses, adjusted for BMI, education and sum score of body pain were performed for associations of TMD myalgia/arthralgia diagnoses with HR and BRS while both standing and sitting for the total sample and stratified by sex (Table 5). BRS while standing was statistically significantly associated with both myalgia and arthralgia diagnoses. In the whole study population, those with myalgia had 17% lower standing BRS compared to the reference group (OR 0.852, 95%Cl 0.763–0.951, *p* = 0.004). Those with arthralgia had 15% lower standing BRS than the reference group (OR 0.866, 95%Cl 0.782–0.964, *p* = 0.005). Among females, standing BRS was 18% lower among those with myalgia compared to those without myalgia (OR 0.847, 95%Cl 0.744–0.964, *p* = 0.012) and 15% lower among those with arthralgia compared to those without arthralgia (OR 0.871, 95%Cl 0.775–0.979, *p* = 0.021).

**TABLE 5:** Association of covariates with myalgia/arthralgia, based on logistic regression, among the total sample of subjects included in the Northern Finland Birth 1966 cohort (*n* = 1895).

Myalgia	OR	95% CI		*p*
		Lower	Upper	
Total				
Overweight	0.622	0.355	1.091	0.098
Obesity	1.002	0.563	1.783	0.996
Basic education	1.133	0.364	3.527	0.830
Higher education	1.467	0.895	2.404	0.129
Pain sum score	1.127	1.090	1.166	**< 0.001**
BRS, standing	0.852	0.763	0.951	**0.004**
Males				
Overweight	1.755	0.347	8.865	0.496
Obesity	3.319	0.603	18.269	0.168
Basic education	1.089	0.116	10.201	0.941
Higher education	1.482	0.481	4.567	0.493
Pain sum score	1.148	1.067	1.236	**< 0.001**
BRS, standing	0.937	0.752	1.166	0.559
Females				
Overweight	0.692	0.365	1.309	0.257
Obesity	0.950	0.503	1.794	0.874
Basic education	1.092	0.289	4.124	0.897
Higher education	1.186	0.673	2.090	0.555
Pain sum score	1.106	1.063	1.150	**< 0.001**
BRS, standing	0.847	0.744	0.964	**0.012**
Arthralgia				
All				
Overweight	0.539	0.319	0.911	**0.021**
Obesity	0.715	0–405	1.263	0.248
Basic education	1.790	0.630	5.082	0.274
Higher education	1.924	1.169	3.166	**0.010**
Pain sum score	1.118	1.081	1.155	**< 0.001**
BRS, standing	0.866	0.782	0.958	**0.005**
Males				
Overweight	1.825	0.364	9.145	0.464
Obesity	3.703	0.679	20.202	0.130
Basic education	1.489	0.165	13.474	0.723
Higher education	1.782	0.582	5.454	0.311
Pain sum score	1.102	1.021	1.189	**0.013**
BRS, standing	0.933	0.751	1.159	0.529
Females				
Overweight	0.600	0.331	1.088	0.093
Obesity	0.610	0.321	1.157	0.130
Basic education	1.894	0.569	6.308	0.298
Higher education	1.602	0.903	2.842	0.107
Pain sum score	1.107	1.065	1.150	**< 0.001**
BRS, standing	0.871	0.775	0.979	**0.021**

Abbreviations: BRS, baroreflex sensitivity; ref. underweight/normal weight; ref. secondary education.

## 4 Discussion

The main finding of the present study was that lower BRS while standing was associated with TMD myalgia or arthralgia diagnoses when adjusted for important covariates among females. As BRS chiefly measures how vagal activity responds to stressors, the present study results may be interpreted to indicate that TMD myalgia and arthralgia are related to disturbed stress response, which was noted especially among females. This suggests higher sympathetic activation among females with pain‐related TMD diagnoses. However, among males, these associations were not significant after adjustment, although in the crude analyses there were also statistically significant differences in HRV variables between those with and without TMD diagnoses in the pair‐wise analyses. The low number of males with TMD diagnosis could explain at least part of this difference in the results. These findings indicate that ANS activity and stress response associate with TMD pain, which is expressed especially among females.

The present results agree with the previous studies concerning TMD and BRS. The OPPERA case–control study, including 185 individuals with TMD diagnoses and 1633 non‐TMD controls, found that both BRS and HRV were reduced among TMD cases, suggesting higher sympathetic tone versus parasympathetic tone [22]. Even though in the present study HRV did not differ statistically significantly between TMD myalgia/arthralgia pain and non‐TMD myalgia/arthralgia pain participants when adjusted for important covariates, HR‐related variables still differed between the groups. Another study from Thailand showed increased sympathetic tone, as indicated by HRV parameters, among TMD patients (*n* = 21) compared to controls (*n* = 23) [23]. Additionally, TMD patients had higher psychological distress and increased salivary cortisol levels. A study by Eze‐Nliam et al. [31] utilised nocturnal polysomnography (PSG) to assess especially HRV in a sample of 37 TMD cases and 58 controls. They found that nocturnal HRV was lower in TMD cases compared to controls.

In the present study, BRS was the most significant ANS variable associated with TMD myalgia/arthralgia. Baroreflex is controlled by both sympathetic and parasympathetic ANS [14]. Individuals with lower BRS have been shown to have decreased short‐term blood pressure regulation and higher sympathetic tone [13]. The connection between higher sympathetic tone and poor general or mental health has been known for a long time [11]. Additionally, in both the OPPERA study and NFBC1966, TMD diagnoses and symptoms have been shown to be associated with poor or fair health condition [5, 7]. Additional studies are needed to investigate whether ANS could act as a mediator between psychosocial factors and TMD.

Autonomic dysfunction may also play a role in the pathophysiology of comorbid conditions linked with TMD and may explain at least some of the mutual connections [32]. TMD patients often suffer from comorbidities, such as multiple pain, headache, pain in the neck, shoulders, back, joints, chronic fatigue syndrome (CFS) and fibromyalgia [33, 34, 35]. Fibromyalgia, CFS, and TMD are thought to be characterised by some degree of ANS dysfunction. Previous results from the NFBC1966 [20] showed that the number of body pain sites associated significantly with BRS among females and with HR standing among males. In the present study, when considering the body pain sum score (indicating the number and frequency of body pain) in the logistic regression analyses, the association between BRS standing and myalgia/arthralgia diagnoses remained significant only among females, indicating their independent role despite the other body pain. This female‐specific association is consistent with prior findings that women tend to show higher parasympathetic reactivity than men, as demonstrated in a large meta‐analysis of HRV indices [36].

It has been suggested that subjects with both TMD and CFS may have increased activity of the parasympathetic nervous system according to HRV measurements. A study by Vuong et al. [37] investigated brain function using functional magnetic resonance imaging (fMRI) while the participants performed the Valsalva manoeuvre, which is a breathing technique activating ANS. The study sample included 52 participants, 26 participants having both CFS and TMD, 16 with CFS without TMD, and 10 age‐matched controls. They found that among those with both CFS and painful TMD, the brain activity related to activation of the autonomic function was higher than among other groups. The explanation for sex differences in the association between TMD diagnoses and BRS may be argued by the findings showing that females are more vulnerable to stress‐related diseases, as compared to men. These differences may be based on potential mechanisms linked to hypothalamus–pituitary–adrenal axis responses, regulation of sex hormones, and immune system responses [38].

The present and previous findings of the association of ANS activity with TMD create a need for studies with interventions affecting the ANS. Previous studies have examined whether increased sympathetic tone affects TMD‐related pain through β_2_‐adrenergic receptor activation when using the beta‐blocker propranolol. For example, one study on FMS and TMD patients (*n* = 54) showed that treatment with low‐dose propranolol resulted in a short‐term symptom improvement in all these domains [39]. However, a larger clinical trial did not find any significant effect of propranolol treatment [40]. The effect of propranolol on TMD pain has been shown especially in the presence of comorbid migraine [40]. Other treatments affecting ANS may include control of stress via relaxation, for instance. Of these, applied relaxation has shown promising results in the treatment of TMD and comorbid pains and unspecific symptoms [41].

The strengths of the present study include the use of valid criteria for TMD, as TMD was diagnosed based on the modified protocol of DC/TMD. The actual DC/TMD criteria [27] were not used because they had not been released at the time of the present study examination. In this study, headache attributed to TMD and myofascial pain with referral were not used separately but were included in TMD myalgia/arthralgia diagnoses. Several cardiovascular autonomic parameters were available concerning HRV and BRS. ANS activity was measured by reliable and valid measures separately from the dental examination. The cohort population was large and representative. Various potential confounders could be considered in the analyses; their selection was based on previous studies which have shown association with TMD [5] and ANS activity [28, 29, 30]. Participants were all the same age; thus, the potential effect of age was controlled. The possible effect of psychosocial factors, such as prolonged stress, was not considered in the present study, which creates a need for further research. The numbers of males with myalgia/arthralgia diagnoses were low (*n* = 20 and 17, respectively). Therefore, one limitation of the study was that the results of the multivariate analysis for males may have included two major types of errors, which require further investigation.

The results of the present study suggest that ANS activity associates with TMD pain, at least to some extent, and it is more obvious among females. Especially, lowered BRS seems to have an association with both TMD myalgia and arthralgia among females. These findings suggest the association of stress response and TMD. There is a need for clinical trials evaluating interventions affecting autonomic function in TMD pain cases.

## Author Contributions

Niklas Kakko main writing of the article and performed the statistical analyses with Auli Suominen. Atte Somero, Mikko Tulppo, Satu Lahti, Vesa Pohjola and Mika Okawa corresponded to the manuscript writing. Kirsi Sipilä supervised the investigation and corresponded to the research plan and manuscript writing.

## Ethics Statement

This study was conducted according to the guidelines of the Declaration of Helsinki (1964). The Ethics Committee of the Northern Ostrobothnia Hospital District approved the research (74/2011, 12 December 2011). All participants gave their informed consent in accordance with the Helsinki Declaration.

## Conflicts of Interest

The authors declare no conflicts of interest.

## Peer Review

The peer review history for this article is available at https://www.webofscience.com/api/gateway/wos/peer‐review/10.1111/joor.14051.

## Data Availability

The data are available from the project center of the Northern Finland Birth Cohort upon request of variables.
